# Transcriptional and metabolic analysis of oleic acid synthesis in seedless and tenera oil palm species

**DOI:** 10.3389/fpls.2025.1557544

**Published:** 2025-02-25

**Authors:** Wen Xu, Jerome Jeyakumar John Martin, Xinyu Li, Xiaoyu Liu, Shunghong Cheng, Hongxing Cao

**Affiliations:** ^1^ College of Tropical Crops, Department of Forestry, Yunnan Agricultural University, Pu’er, China; ^2^ Coconut Research Institute, Chinese Academy of Tropical Agricultural Sciences, Wenchang National Key Laboratory for Tropical Crop Breeding, Haikou, Wenchang, China

**Keywords:** oil palm, oleic acid biosynthesis, transcriptome, metabolome, lipid metabolism

## Abstract

The oil palm (*Elaeis guineensis* Jacq.) is a perennial oilseed crop whose mesocarp produces palm oil rich in the unsaturated fatty acid oleic acid, known for its oxidative stability and cardiovascular health benefits. However, the regulatory mechanisms and pathways responsible for variations in oleic acid biosynthesis during fruit development remain inadequately elucidated. The study examined the mesocarp of oil palm fruits from three developmental stages in seedless and Tenera varieties to evaluate oleic acid content. Fruits from Seedless (MS) and Tenera (MT) oil palms, pollinated for 95 days (MS1 and MT1), 125 days (MS2 and MT2), and 185 days (MS3 and MT3), were analyzed using metabolomics via liquid chromatography-tandem mass spectrometry (LC-MS/MS). RNA sequencing was conducted to profile gene expression associated to oleic acid biosynthesis and accumulation. Differential genes and metabolites were mapped and functionally enriched through KEGG pathway analysis. The result revealed that *SAD*, *FabD*, *LACS6*, *BC*, *FabB*, and *FabI* were positively associated with oleic acid content, whereas *LACS9* exhibited either a negative or strongly negative correlation. By integrating metabolomic and transcriptomic techniques, this study elucidates the distinct mechanisms of oleic acid biosynthesis in seedless and thin-shelled oil palm varieties. These findings provide a scientific foundation for enhancing oleic acid content and improving the quality of oil palm-derived products.

## 1 Introduction

The oil palm (*Elaeis guineensis* Jacq.) is one of the most important oil crops globally, renowned for its superior productivity compared to other oilseed crops. Its oil yield is approximately eight to ten times greater than that of soybean and rapeseed, respectively, making it the most productive oil crop worldwide (Papilo et al., 2022). The mesocarp of oil palm fruit is the primary oil-producing tissue, containing predominantly 38–45% palmitic acid (C16:0) and 38–44% oleic acid (C18:1) (Dussert et al., 2013; Morcillo et al., 2021).

Oleic acid, a monounsaturated fatty acid, is known for its exceptional oxidative stability, which supports immune regulation and helps prevent cardiovascular diseases (Katariya et al., 2021). After ingestion, oleic acid undergoes hydrolysis in the stomach and enters the bloodstream intact as glycerol monoesters (Voon et al., 2019). Moreover, studies suggest that high-oleic oil palm can generate an estimated economic value of $1,500 per hectare annually when oleic acid content exceeds 65% (Sambanthamurthi et al., 2002). In comparison, high-oleic acid rapeseed oil typically contains 75–84% oleic acid (Chang et al., 2022). As a result, oleic acid holds significant nutritional and economic value. Consequently, the developing oil palm varieties with elevated oleic acid content through genetic enhancement has become a critical objective in breeding programs (Bahariah et al., 2021).

Previous research has identified key enzyme genes involved in oleic acid synthesis in oil palm. Sun et al. (2016) successfully cloned Δ12-desaturase, a gene associated with the biosynthesis of long-chain polyunsaturated fatty acids (LC-PUFAs), and demonstrated that *egFAD12* exhibited peak activity in oil palm fruits 120–140 days after pollination. This enzyme facilitates the conversion of oleic acid to linoleic acid. Ben Ayed et al. (2022) performed a comparative analysis of 24 *FAD2* sequences from different species and identified two single nucleotide polymorphisms (SNPs), SNP373 and SNP718, associated with oleic and linoleic acid content. These SNPs were shown to regulate *FAD2* activity, either reducing or increasing oleic acid levels. Additionally, Bahariah et al. (2023) utilized a multiplex CRISPR/Cas9 platform to successfully induce mutations in the *EgFAD2* gene, located on chromosome 8, and the *EgPAT* gene (also known as *EgFATB-1*), found on chromosomes 3 and 7. This approach aims to reduce the activity of the *PAT* and *FAD2* enzymes, thereby enhancing oleic acid content. To achieve high levels of 18:1 for improved oil stability and human health, *FAD2* has become a prime target for disruption using the powerful genome editing tool CRISPR/Cas9 (Okuzaki et al., 2018; Al Amin et al., 2019; Do et al., 2019). Wei et al. (2024) further identified six enzyme genes—*BC*, *ACC*, *FabB*, *FabI*, *FabG*, and *FabD*—that promote oleic acid accumulation during oil palm fruit development.

An AGAMOUS-like MADS-box transcription factor, *EgAGL9*, was identified through expression profiling at various developmental stages of the oil palm pericarp. Research by Zhang et al. (2022) demonstrated that *EgAGL9* interacts with the enzyme genes *EgSAD*, *EgTSA*, and *EgSDH* in the fatty acid synthesis pathway, leading to a substantial reduction in the proportion of unsaturated fatty acids, including oleic, linoleic, and linolenic acids. Additionally, *EgMADS2* has been shown to regulate *EgDGAT* expression, significantly decreasing linoleic acid content while increasing oleic acid levels in transgenic embryos (Li et al., 2020). A full-length cDNA encoding *EgLACS9* was also cloned from oil palm pericarp. Overexpression of *EgLACS9* in the *LACS*-deficient *Saccharomyces cerevisiae* strain YB525 resulted in reduced levels of C18:1 (oleic acid) and C18:2 (linoleic acid) (Wang et al., 2021). Moreover, downregulation of the transcription factor *EgGRP2A* resulted in decreased expression of *EgFATA* and a concomitant reduction in oleic acid content (Luo et al., 2024). A seed-like fruit-specific complex network has been established in the mesocarp of oil palm.NF-YA3, NF-YC2, and ABI5 directly activate WRI1-1 and a subset of FA synthesis genes. NF-YA3 also physically interacts with NF-YC2, ABI5 and WRI1-1 to form transcription complexes that regulate gene expression. WRKY40 cooperates with WRKY2 to repress ABI5, resulting in the synthesis of oils and oils (Yeap et al., 2017). Additionally, researchers have explored the biosynthesis, transport, key enzymes and their regulation of C18 UFAs, especially the emerging regulatory network involving transcription factors and upstream signaling pathways (He et al., 2020). The findings collectively contribute to understanding the genetic regulation of oleic acid synthesis and highlight potential targets for genetic improvement to enhance oil quality.

Although initial investigations into oleic acid in oil palm have been conducted, there is a lack of comprehensive studies examining the variation in oleic acid content across different oil palm varieties and developmental stages. This study utilized high-throughput sequencing technology for transcriptomics and liquid chromatography-tandem mass spectrometry (LC-MS/MS) for metabolomics to examine the changing patterns of oleic acid during oil palm growth and development. By analyzing the differences in oleic acid content across various oil palm varieties, this research elucidates the dynamic characteristics of oleic acid at different developmental stages and provides a scientific foundation for enhancing the quality of oil palm.

## 2 Materials and methods

### 2.1 Experimental materials

The experimental samples were collected from oil palm fruits of seedless and thin-shelled varieties, harvested at different developmental stages from the Coconut Research Institute of the Chinese Academy of Tropical Agricultural Sciences (19°33′N, 110°47′E), located at the institute’s oil palm test site. The samples consisted of mesocarp tissue obtained from oil palm fruits at three distinct developmental stages: 95 days (early developmental stage: MS1 and MT1), 125 days (mid-developmental stage: MS2 and MT2), and 185 days (late developmental stage: MS3 and MT3) post-pollination. For each developmental stage, three biological replicates were taken, ensuring a robust representation of the variation within each stage. Following collection, all samples were immediately stored in liquid nitrogen at -80°C to preserve metabolomic and transcriptomic integrity for subsequent analysis. These stored samples were then subjected to high-throughput sequencing and liquid chromatography-tandem mass spectrometry (LC-MS/MS) for in-depth investigation of the metabolic and transcriptional profiles.

### 2.2 Metabolomics analysis and data processing

Mesocarp samples were prepared for metabolomic analysis using an ExionLC ultra-high-performance liquid chromatography (UHPLC) system coupled with a SCIEX QTRAP 6500+ tandem mass spectrometer. Metabolite extraction involved tissue homogenization, solvent extraction, and filtration to ensure sample integrity and reproducibility. Metabolite separation was conducted under optimized chromatographic conditions, and detection was performed in multiple reaction monitoring (MRM) mode for high sensitivity and specificity. Raw mass spectrometry data were processed with Analyst 1.6.3 software, including peak detection, alignment, and integration. Metabolite identification was achieved by matching spectral data with local lipid databases, while spectral peaks across samples were aligned and corrected for variability. Absolute metabolite concentrations were determined from integral peak areas. Differentially abundant metabolites were identified through orthogonal partial least squares discriminant analysis (OPLS-DA), with selection criteria including a variable importance in projection (VIP) score of ≥ 1, a fold change of ≥ 2 for upregulation, or ≤ 0.5 for downregulation. Results from MRM mode were visualized in **Supplementary Figure S2**, and identified metabolites were further analyzed to provide insights into oleic acid biosynthesis, elucidating developmental and varietal differences in oil palm.

### 2.3 Mesocarp RNA extraction and high throughput sequencing

Total RNA was extracted from mesocarp tissues collected at 95, 125, and 185 days after pollination using a commercial RNA isolation kit (Tiangen Biotech, China), following the manufacturer’s protocol, with homogenization performed under liquid nitrogen to preserve RNA integrity. RNA quality and integrity were assessed using an Agilent 2100 Bioanalyzer, with samples achieving RNA integrity number (RIN) values ≥ 7.0 deemed suitable for downstream applications. Purity was evaluated using A260/A280 and A260/A230 ratios measured on a NanoDrop spectrophotometer, while RNA concentrations were quantified with a Qubit RNA assay kit. The cDNA fragments underwent end repair, A-tailing, adapter ligation, and PCR amplification, with library quality assessed using an Agilent 2100 Bioanalyzer and concentrations determined via qPCR. After passing the library check, different libraries are pooled according to the effective concentration and the target downstream data volume required for Illumina sequencing, and 150bp paired-end reads are generated. Four types of fluorescently labeled dNTP, DNA polymerase, and junction primers are added to the sequencing flow cell for amplification. When extending the complementary strand of each sequencing cluster, each fluorescently labeled dNTP added releases corresponding fluorescence, which is then captured by the sequencer, and converted into sequencing peaks by the computer software, thus obtaining the sequence information of the fragment to be sequenced.

Raw data were filtered using fastp v 0.19.3, mainly removing reads with adapters; when the N content of any sequencing read exceeded 10% of the number of bases in the read, the paired reads were removed; when the number of low-quality (Q<=20) bases in any sequencing read exceeded 50% of the number of bases in the read, the paired reads were removed. All subsequent analyses were based on clean reads. reference genomes and their annotation files were downloaded from the indicated websites, indexes were constructed using HISAT v2.1.0, and clean reads were aligned to the reference genomes. Differentially expressed genes (DEGs) were identified using DESeq2 with stringent criteria: false discovery rate (FDR) < 0.05 and |log2FoldChange| ≥ 1. Identified DEGs underwent further analysis, including Nr functional annotation, enrichment analysis, and other bioinformatics approaches, to explore their roles in metabolic and regulatory pathways.

### 2.4 Statistical analysis

Data analysis was performed using Excel 2019 and SPSS software. For graphical representation, Origin 2022 and GraphPad Prism 9.5 were utilized. Venn diagrams and heatmaps were generated using the online tool available at https://www.omicshare.com/tools/. Statistical significance was determined based on the appropriate tests for each data set, with p-values < 0.05 considered statistically significant.

## 3 Results and analyses

### 3.1 Analysis of the dynamics of oleic acid metabolites in the pulp of seedless and thin-shelled oil palms at different stages of development

**Figure 1:**
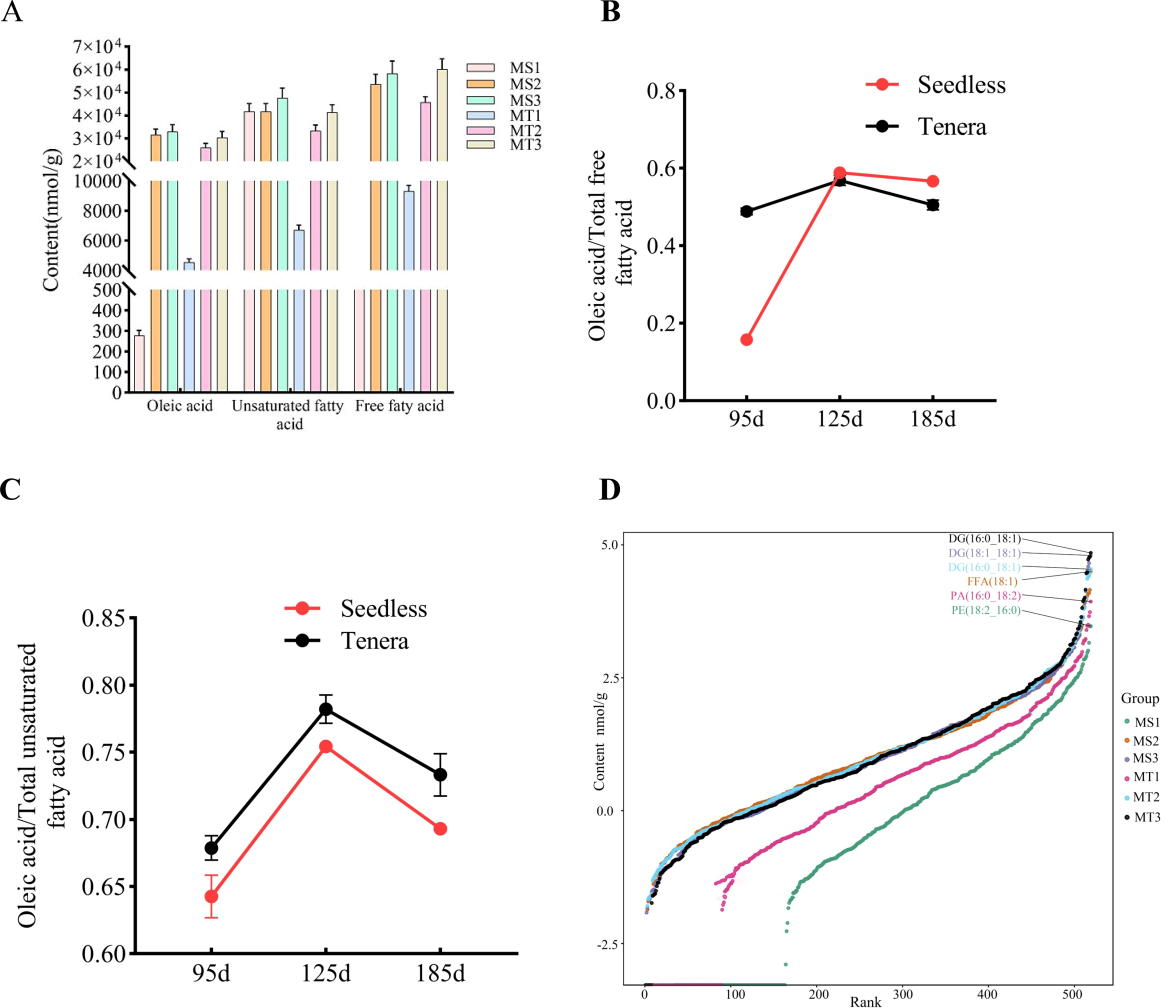
Dynamic changes of oleic acid content in Seedless and Tenera oil palm at different developmental stages. **(A)** Dynamics of oleic acid, unsaturated fatty acids, and total free fatty acids **(B)** Oleic acid as a percentage of total free fatty acids **(C)** Oleic acid as a percentage of total unsaturated fatty acids **(D)** Dynamic distribution of substances in all substance groups. Each point in the graph represents a lipid molecule. The vertical coordinate represents the corresponding content (log10 conversion) of each lipid molecule, and the lipid molecules with the highest contents are labelled. Different color curves represent different groupings.

Studies on oleic acid metabolites in the pulp of seedless and thin-shelled oil palm varieties at various developmental stages revealed that the free fatty acid composition in the pulp consisted of 13 unsaturated and 17 saturated fatty acids, with oleic acid being the dominant fatty acid. In the seedless oil palm variety, oleic acid content gradually increased from 277.30 nmol/g at the MS1 stage to 31,536.14 nmol/g at the MS2 stage, reaching a peak of 32,992.80 nmol/g at the MS3 stage. This increase represented 15.64%, 58.81%, and 56.66% of the total free fatty acid content at each respective stage. Furthermore, oleic acid accounted for 64.27%, 75.44%, and 69.31% of the total unsaturated fatty acids at these stages (**Figures 1A–C**).

In the thin-shelled oil palm variety, oleic acid content increased from 4,549.25 nmol/g at the MT1 stage to 26,021.06 nmol/g at the MT2 stage, reaching a maximum of 30,396.86 nmol/g at the MT3 stage. This corresponded to 48.85%, 56.88%, and 50.51% of the total free fatty acid content, respectively. Oleic acid also made up 68.87%, 78.2%, and 73.31% of the total unsaturated fatty acids at the respective stages (**Figures 1A–C**).

The increase in oleic acid content from the MT1 to MT2 stage was greater in seedless oil palm (31,258.84 nmol/g) than in thin-shelled oil palm (21,471.81 nmol/g). From mid-development onwards, the oleic acid content of seedless oil palm exceeded that of thin-shelled oil palm, and the oleic acid content of the two varieties of oil palm reached a maximum at a later stage of development, a trend reflecting the pattern of unsaturated fatty acid content of the two varieties. The contribution of lipids in the pulp of the two varieties of oil palm at different developmental stages is as follows: at the early stage of development of the seedless species (MS1), the highest content of phosphatidylethanolamine (18:2_16:0), at the middle stage of development of the fruit (MS2), the highest content of fatty acids in the fruit pulp is said to be oleic acid (18:1), and at the late stage of development (MS3), the highest content of glycerol diesters (18:1_18:1). MS3), the highest content in the pulp was triglycerides (18:1_18:1); in the early stage of development of thin-shelled species of oil palm (MT1), the highest content in the pulp was phosphatidic acid (16:0_18:2), and in the middle stage of development of thin-shelled species of oil palm (MT2) and the late stage of development (MT3), the highest content in the pulp was triglycerides (16:0_18:1) (**Figure 1D**).

### 3.2 Differential metabolite analysis of comparative groups across developmental periods

**Figure 2:**
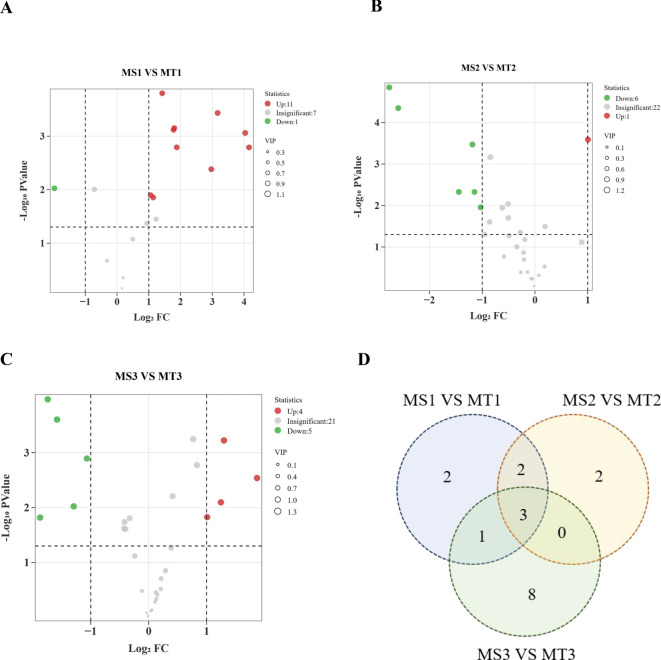
Statistics of different metabolites of oil palm in Seedless and Tenera **(A-C)**. **(A-C)** are volcano plots of MS1 VS MT1, MS2 VS MT2 and MS3 VS MT3 differential metabolites, respectively **(D)** Venn map. Each point represents a metabolite, with the horizontal axis showing the logarithm of the fold change between the two sample varieties (Log2 fold change) and the vertical axis representing the significance of the variable (VIP). Larger absolute values on the horizontal axis indicate greater significance of the difference. Green dots denote down-regulated metabolites, red dots represent up-regulated metabolites, and gray dots indicate metabolites with significant changes.

Differential metabolite analysis was conducted across different developmental periods of oil palm, using criteria of fold-change ≥ 2 or fold-change ≤ 0.5 and VIP ≥ 1. A total of 19 differential metabolites were identified through comparisons of three developmental stages of oil palm (**Table 1**). In the comparison between the MS1 vs. MT1 stage, 12 differential metabolites were found, consisting of 11 up-regulated metabolites and 1 down-regulated metabolite (**Figure 2A**). At the MS2 vs. MT2 stage, 7 differential metabolites were identified, with 1 up-regulated metabolite and 6 down-regulated metabolites (**Figure 2B**). Finally, at the MS3 vs. MT3 stage, 9 differential metabolites were observed, with 4 up-regulated and 5 down-regulated metabolites (**Figure 2C**).

**Table 1:** Differential metabolites in different comparison groups of oil palm mesocarp in Seedless and Tenera oil palm.

Comparable Group	Lipid Metabolites ID	Up/Down
MS1 VS. MT1	Lipid-B-N-0030; Lipid-B-N-0051; Lipid-B-N-0039; Lipid-B-N-0034;Lipid-B-N-0028; Lipid-B-N-0026; Lipid-B-N-0013; Lipid-B-N-0008; Lipid-B-N-0007; Lipid-B-N-0006; Lipid-B-N-0005	Up
	Lipid-B-N-0017	Down
MS2 VS. MT2	Lipid-B-N-0005	Up
	Lipid-B-N-0010;Lipid-B-N-0026;Lipid-B-N-0019;Lipid-B-N-0017;Lipid-B-N-0003;Lipid-B-N-0050	Down
MS3 VS. MT3	Lipid-B-N-0010;Lipid-B-N-0013;Lipid-B-N-0005;Lipid-B-N-0012	Up
	Lipid-B-N-0027;Lipid-B-N-0029;Lipid-B-N-0026;Lipid-B-N-0019;Lipid-B-N-0017	Down

Three metabolites were commonly differential across all three comparison groups (**Figure 2D**): tridecanoic acid (Lipid-B-N-0017), myristic acid (Lipid-B-N-0005), and palmitoleic acid (Lipid-B-N-0026) (**Table 2**). Among these, only myristic acid was consistently up-regulated across all stages, while tridecanoic acid was the only metabolite consistently down-regulated. Palmitoleic acid was up-regulated in the early developmental stages but down-regulated in the mid- and late stages. Additionally, oleic acid (Lipid-B-N-0028) was up-regulated during the early stages of development. These findings offer valuable insights into the dynamic changes of fatty acid metabolites in the pulp of oil palm across different developmental stages.

**Table 2:** Detailed information on common differential metabolites in the three comparison groups.

Lipid Metabolites ID	Lipid Metabolites	Q1 (Da)	Molecular Weight	Ionization model	Formula
Lipid-B-N-0026	FFA(16:1)	253.216755	254.22458	[M-H]-	C16H30O2
Lipid-B-N-0017	FFA(30:0)	451.44965	368.36543	[M-H]-	C24H48O2
Lipid-B-N-0005	FFA(14:0)	227.201105	228.20893	[M-H]-	C14H28O2

### 3.3 Analysis of significantly differentially expressed genes at different developmental periods

**Figure 3:**
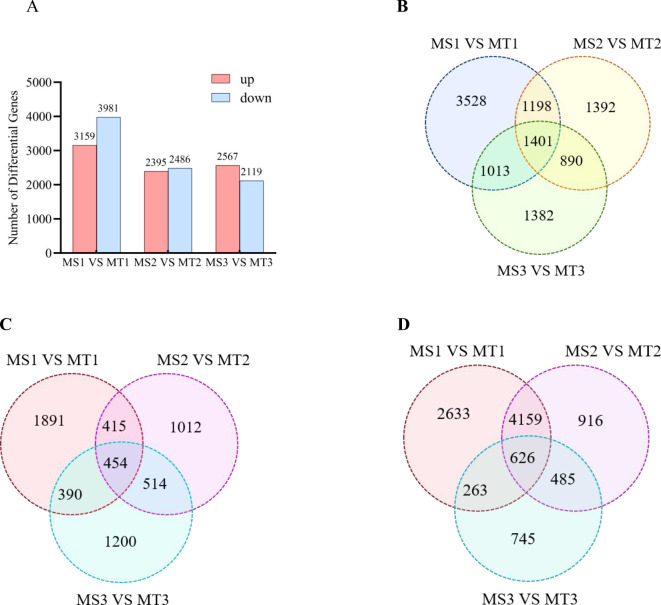
Statistics of different genes of oil palm in Seedless and Tenera **(A)** Histogram **(B)** Venn map of up/down-regulated differential genes **(C)** Venn map of up-regulated differential genes **(D)** Venn map of down-regulated differential genes.

Analysis of differentially expressed genes (DEGs) across developmental periods in oil palm revealed significant up- and down-regulation of gene expression. A comparison of transcriptome data between the two oil palm varieties at each growth stage during three developmental periods (MS1 vs. MT1, MS2 vs. MT2, and MS3 vs. MT3) identified 7140, 4881, and 4686 DEGs, respectively. Of these, 3159, 2395, and 2567 were up-regulated, while 3991, 2491, and 2129 were down-regulated (**Figure 3A**). Across all three comparative groups, a total of 1401 genes were significantly differentially expressed (**Figure 3B**). Additionally, 454 up-regulated and 626 down-regulated genes were common across both oil palm varieties in the three developmental periods (**Figures 3C, D**).

### 3.4 Enrichment analysis of significantly differentially expressed genes of oil palm fruits at different developmental periods

**Figure 4:**
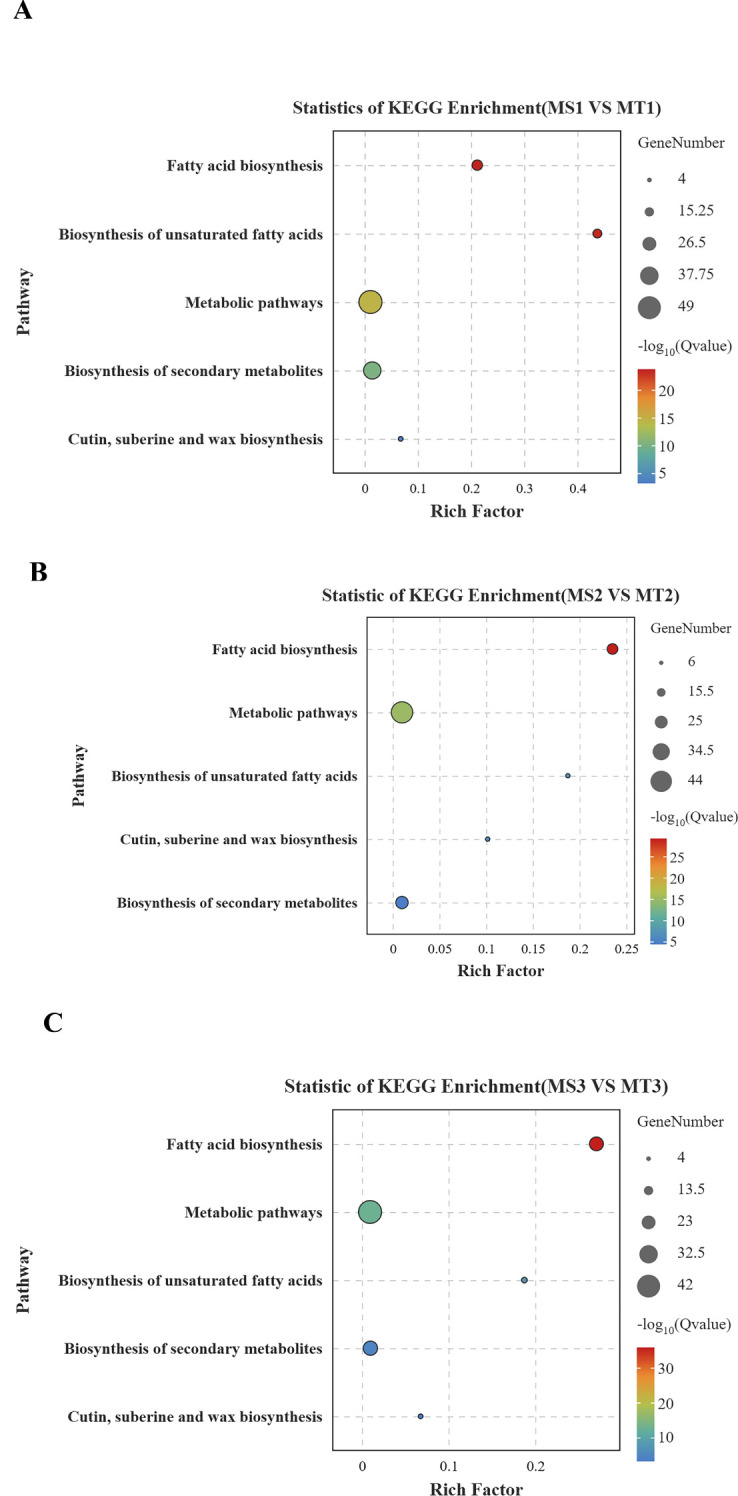
Bubble map of KEGG enrichment in oil palm fruit at different developmental periods. **(A)** differential gene KEGG results for MS1 vs MT1; **(B)** differential gene KEGG results for MS2 vs MT2; **(C)** differential gene KEGG results for MS3 vs MT3. The size of the circle indicates the number of different genes, and the larger the circle, the more genes. q value is a p-value that has been verified by multiple factors.

Enrichment analysis of significantly differentially expressed genes (DEGs) in oil palm fruits across different developmental periods, integrating metabolome data with transcriptome data and referencing KEGG metabolic pathway information, revealed that free fatty acids were enriched in five key lipid metabolism pathways of oil palm (**Figures 4A–C**). The five fatty acids with significant differences in the fatty acid biosynthesis pathways of oil palm, including those from Seedless and Tenera species, were two unsaturated fatty acids—oleic acid and palmitoleic acid—and three saturated fatty acids—palmitic acid, lauric acid, and myristic acid. These fatty acids corresponded to 37 differential genes (**Table 3**).

**Table 3:** Differential lipid metabolites and gene statistics of fatty acid biosynthesis (ko00061) pathway at different developmental periods of Seedless and Tenera oil palm mesocarp.

Comparable group	Lipid Metabolites ID	Lipid Metabolites	Genes ID
MS1 VS MT1	Lipid-B-N0028	FFA(18:1)Oleic acid	LOC105034511;LOC105035520;LOC105039456;LOC105042279;LOC105057927;LOC105061240;LOC105061231;LOC105040700;LOC105044978;LOC105048199;LOC105051934;novel.3082;LOC105050555;LOC105037839;LOC105049670;LOC105051176;novel.3968;LOC105050310
	Lipid-B-N0026	FFA(16:1) Palmitoleic acid	
	Lipid-B-N0007	FFA(16:0) Palmitic acid	
	Lipid-B-N-0005	FFA(14:0) Myristic acid	
MS2 VS MT2	Lipid-B-N0026	FFA(16:1) Palmitoleic acid	LOC105034507;LOC105035520;LOC105042279;LOC105042280;LOC105060936;LOC105058955;LOC105035642;LOC105039221;LOC105038382;LOC105040698;LOC105040700;novel.3082;LOC105050555;ElguCp049;LOC105051176;LOC105042027;LOC105056688;LOC105047747;LOC105050310;LOC105048939
	Lipid-B-N0005	FFA(14:0) Myristic acid	
	Lipid-B-N-0003	FFA(12:0) Lauric acid	
MS3 VS MT3	Lipid-B-N-0026	FFA(16:1) Palmitoleic acid	LOC105034507;LOC105034511;LOC105035520;LOC105039456;LOC105042279;LOC105061231;LOC105035641;LOC105040698;LOC105040700;LOC105044978;novel.3082;LOC105049274;ElguCp049;LOC105058068;LOC105037839;LOC105042027;LOC105056688;novel.2764;LOC105047747;LOC109506086;PTE;LOC105050310;LOC105048939
	Lipid-B-N-0005	FFA(14:0) Myristic acid	

**Figure 5:**
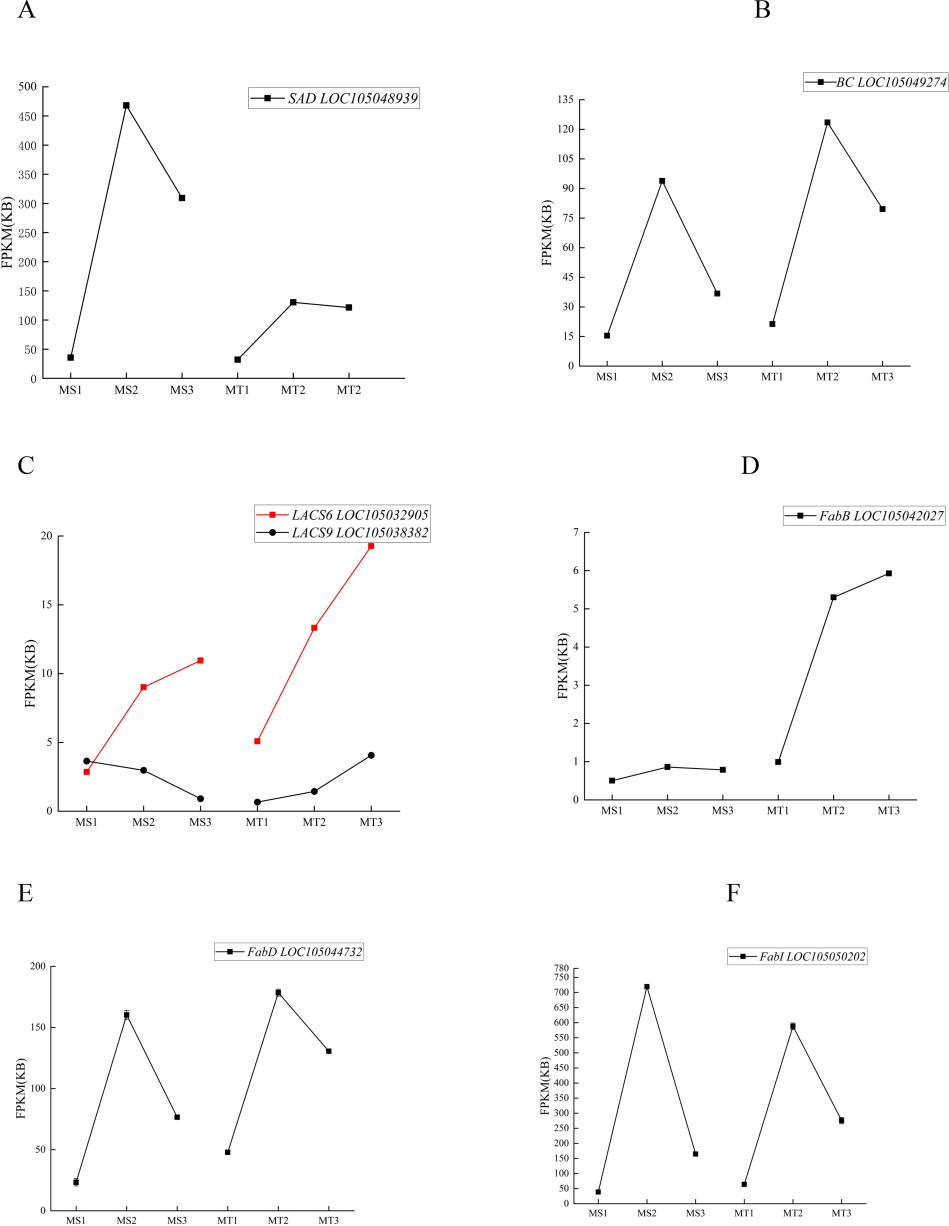
The expression levels of key enzyme genes changed dynamically in different developmental stages of seedless and Tenera oil palm **(A-F)**.

NR annotation of the 37 significantly differentially expressed genes in Seedless and Tenera oil palm species highlighted that genes such as *SAD、 FabD*, *LACS6*, *LACS9*, *FabB*, *BC*, and *FabI* were highly expressed during oil palm fruit development. Regarding the dynamic changes in the expression of these enzyme genes, *SAD* and *BC* exhibited a trend of increasing and then decreasing expression during fruit development in both seedless and thin-shelled oil palms, peaking at the middle developmental stage (**Figures 5A, B**). *LACS6* expression increased and peaked at the late developmental stage, while *LACS9* expression patterns differed between the two varieties: in seedless oil palm, *LACS9* expression was highest at MS1 and then decreased, whereas in thin-shelled oil palm, *LACS9* expression was lowest at MT1, subsequently increased, and peaked at MT3 (**Figure 5C**). *FabB* expression increased with development in thin-shelled oil palm and peaked at MT3 (**Figure 5D**), and the expression patterns of *FabD* and *FabI* increased and then decreased, reaching a maximum during the middle developmental stage (**Figures 5E, F**).

The dynamic changes in the content of the five differential metabolites were correlated with the expression changes of these seven enzyme genes (**Figure 5**; **Tables 4**, **5**). The results indicated that *SAD*, *FabD*, *LACS6*, *BC*, *FabB*, and *FabI* were positively correlated with oleic acid content in both oil palm varieties, while *LACS9* exhibited a negative correlation with these five free fatty acids (**Tables 4**, **5**). This suggests that the expression of these enzyme genes (*SAD*, *FabD*, *LACS6*, *BC*, *FabB*, and *FabI*) promotes oleic acid accumulation in the pulp, while *LACS9* expression inhibits this accumulation.

**Table 4:** Correlation analysis of fatty acid content and enzyme genes in Seedless oil palm.

Gene ID	Enzyme name	Oleic acid	Myristic Acid	Palmitic acid	Palmitoleic acid	Lauric acid
*LOC105044732*	*FabD*	0.762	0.840	0.843	0.609	0.707
*LOC105032905*	*LACS6*	0.956	0.916	0.911	0.979	0.763
*LOC105038382*	*LACS9*	-0.973	-0.966	-0.965	-0.937	-0.693
*LOC105049274*	*BC*	0.677	0.771	0.775	0.505	0.663
*LOC105048939*	*SAD*	0.912	0.956	0.959	0.807	0.809
*LOC105047027*	*FabB*	0.714	0.801	0.806	0.550	0.687
*LOC105050202*	*FabI*	0.607	0.710	0.715	0.425	0.617

**Table 5:** Correlation analysis of fatty acid content and enzyme genes in Tenera oil palm.

Gene ID	Enzyme name	Oleic acid	Myristic Acid	Palmitic acid	Palmitoleic acid	Lauric acid
*LOC105044732*	*FabD*	0.854	0.666	0.733	0.689	-0.940
*LOC105032905*	*LACS6*	0.952	0.994	0.994	0.982	-0.674
*LOC105038382*	*LACS9*	-0.946	-0.981	-0.985	-0.959	0.644
*LOC105049274*	*BC*	0.820	0.611	0.682	0.638	-0.934
*LOC105048939*	*SAD*	0.951	0.848	0.890	0.858	-0.911
*LOC105047027*	*FabB*	0.880	0.718	0.777	0.736	-0.943
*LOC105050202*	*FabI*	0.693	0.454	0.532	0.488	-0.904

## 4 Discussion

The study revealed a significant increase in oleic acid levels during the developmental stages of oil palm fruits in both seedless and thin-shelled varieties, with pronounced accumulation occurring in the middle stage. By the late stage, the oleic acid content stabilized. In the late developmental stage, oleic acid content stabilized. Throughout the developmental process, the seedless species consistently exhibited higher oleic acid levels compared to the thin-shelled species. These results provide a foundational basis for the development of high-oleic-acid planting materials and strategies to enhance oleic acid content.

### 4.1 *LACS* family genes exhibit distinct roles in oleic acid biosynthesis

Previous research supports these findings, highlighting the conserved roles of *LACS* family genes across different plant species. In Arabidopsis thaliana, most of the *LACS* family genes (comprising nine members) have been well characterized, with distinct functions in lipid metabolism (Zhang et al., 2018). Notably, *LACS6* in Arabidopsis has been implicated in the activation of fatty acids for storage lipid synthesis, aligning with our observations in oil palm. Additionally, studies in other crops have revealed similar patterns. For instance, two *LACS6* enzyme genes were found to exhibit strong correlations between their expression levels and metabolite content in oil-producing tissues. Specifically, *LACS6* showed high expression in the pulp, with peak expression occurring during the mid-to-late developmental stages (MT3 period), which is consistent with our findings in oil palm. Similar trends were also reported in stalked lentils (Bao et al., 2021), where *LACS6* expression was closely associated with increased lipid accumulation during key developmental phases. Zhong et al. (2023) reported that the expression of nearly all *LACS* genes was significantly higher in high-seeded oilseed cotton compared to low-seeded varieties, highlighting their importance in seed oil production. Similarly, in kale-type oilseed rape, *BnLACS2* plays a crucial role in seed oil biosynthesis, exhibiting substrate preferences for fatty acids such as 14:0, 16:0, 18:0, 18:1, and 22:1 (Ding et al., 2020). Furthermore, *LACS* genes have been shown to exhibit distinct expression patterns across different tissues and developmental stages (Ayaz et al., 2021). For instance, earlier research found that overexpression of *EgLACS9* in oil palm led to a significant reduction in palmitic, oleic, and linoleic acids (Wang et al., 2021), which is consistent with our observation that LACS9 may inhibit oleic acid accumulation in seedless oil palms. In contrast, *LACS6* has been identified as key enzyme in activating long-chain fatty acids for cellular lipid synthesis and degradation through β-oxidation (Shockey et al., 2002). In the present study, the *LACS6* exhibited a positive correlation with the five major free fatty acids in seedless oil palm (**Table 4**), indicating its role in enhancing the synthesis of these fatty acids in this variety. However, in thin-shelled oil palm, *LACS6* showed a negative correlation with lauric acid but positive correlation with palmitic acid, oleic acid, palmitoleic acid, and myristic acid (**Table 5**). This suggests that *LACS6* may promote the synthesis of these four fatty acids while inhibiting the lauric acid production in the mesocarp of thin-shelled oil palm.

### 4.2 *BC* genes promote oleic acid production

The *BC* gene plays an important role in fatty acid biosynthesis by catalyzing the biotin carboxylation reaction (Laseke et al., 2023). In the present study, *BC* exhibited a positive correlation with the levels of five free fatty acids in the pulp of seedless oil palm (**Table 4**), suggesting that *BC* may facilitate the synthesis of these fatty acids in this variety. Interestingly, in thin-shelled oil palm, ***BC*** showed a contrasting pattern- it was negatively correlated with lauric acid but positively correlated with palmitic acid, palmitoleic acid, oleic acid, and myristic acid (**Table 5**). This indicates that the expression of the *BC* enzyme gene may enhance the synthesis of the four fatty acids while simultaneously inhibiting the production of lauric acid in thin-shelled oil palm.

### 4.3 Role of *SAD* genes in promoting oleic acid biosynthesis

*SAD* genes play a pivotal role in fatty acid biosynthesis by catalyzing the desaturation of stearic acid (C18:0) to oleic acid (C18:1), a key step in the production of unsaturated fatty acids (Liu et al., 2020). In hybrid oil palm, the expression of *OeSAD1*, *OeSAD2*, and *OeSAD3* has been observed to increase during early developmental stages before declining in later stages, highlighting their dynamic role in lipid metabolism (Wang et al., 2022). Similarly, in peony seeds, *SAD1* and *SAD2* have been shown to catalyze the desaturation of C18:0 to C18:1, underscoring their importance in the biosynthesis of unsaturated fatty acids (Li et al., 2021). In the present study, *SAD* genes exhibited a positive correlation with palmitic acid, palmitoleic acid, oleic acid, myristic acid, and lauric acid in the pulp of seedless oil palm (**Table 4**). This suggests that *SAD* may promote the synthesis of these fatty acids during fruit development in seedless varieties. However, in thin-shelled oil palm, *SAD* genes showed a positive correlation with palmitic acid, palmitoleic acid, oleic acid, and myristic acid, but a negative correlation with lauric acid (**Table 5**). This indicates that *SAD* may enhance the synthesis of the former four fatty acids while suppressing lauric acid production in thin-shelled varieties. The result suggest that the dual role of *SAD* genes in regulating fatty acid composition, depending on the oil palm variety and developmental stage. The positive correlation between *SAD* and oleic acid in both seedless and thin-shelled oil palms underscores its critical role in promoting oleic acid biosynthesis. At the same time, the contrasting effects on lauric acid production suggest that *SAD* may act as a regulatory switch, modulating the balance between saturated and unsaturated fatty acids.

### 4.4 *FabB*, *FabD*, and *FabI* genes drive oleic acid accumulation in oil palm mesocarp

The *FabB*, *FabD*, and *FabI* genes play critical roles in the fatty acid biosynthesis pathway, contributing significantly to the accumulation of oleic acid in the mesocarp of oil palm. *FabB* acts as an activator of fatty acid synthase (FAS) and functions as a β-oxoacyl-ACP synthase, catalyzing the addition of acyl-ACP to malonyl-ACP to form β-oxoacyl-ACP, a key step in fatty acid synthesis II pathway (Lee et al., 2013). Enzymes encoded by the *FabA* and *FabB* genes are known to introduce double bonds in decane precursors, which are elongated into unsaturated fatty acyl chain such as the 16:1Δ9 and 18:1Δ11 essential for functional membrane phospholipids (Wei et al., 2024). In microalgae *fabB/F* has been identified as playing a key role in the elongation of medium fatty acids (C < 18) and the conversion of cis-16:1 to cis-18:1 (Gong and Miao, 2019). In this study, *FabB* was positively correlated with palmitic acid and myristic acid, oleic acid, and lauric acid in the pulp of seedless oil palm (**Table 4**). In thin-shelled oil palm, *FabB* showed positive correlation with palmitic acid, myristic acid, oleic acid, and palmitoleic acid but a negative correlation with lauric acid (**Table 5**). The results suggest that *FabB* supports the production of palmitic acid, myristic acid, and oleic acid in both seedless and thin-shelled oil palm varieties, while also enhancing lauric acid synthesis in seedless species and inhibiting its formation in thin-shelled species during fruit development.

*FabD* is a crucial enzyme in the type II fatty acid synthesis pathway, responsible for transferring malonyl groups from malonyl coenzyme A to acyl carrier protein (ACP) to form malonyl-ACP and free coenzyme A-SH (Magnuson et al., 1993). In this study, *FabD* was positively correlated with the levels of five free fatty acids in the pulp of seedless oil palm (**Table 4**), and with oleic acid content in the pulp of Tenera oil palm (**Table 5**). The results suggest that the *FabD* likely facilitates oleic acid synthesis in both seedless and thin-shelled oil palm species.

*FabI* catalyzes the final step in type II fatty acid biosynthesis and is widely utilized for the production of pharmacologically relevant chiral intermediates, such as palmitic and oleic acids, due to its unique properties in reduction and asymmetric enantioselective catalysis (Zhou et al., 2023). In this study, the *FabI* was positively correlated with palmitic and oleic acid content in the pulp of seedless oil palm (**Table 4**). Additionally, *FabI* showed a strong positive correlation with oleic acid in the pulp of thin-shelled oil palm, with a notably high expression levels during the middle stage of development (**Table 5**). These findings suggest that the *FabI* plays a significant role in promoting oleic acid synthesis.

## 5 Conclusion

Significant differences in oleic acid content were observed between seedless and thin-shelled oil palms at various developmental stages. During the early developmental period, oleic acid content was higher in thin-shelled oil palms compared to seedless species. This difference may be attributed to the high expression of *LACS9* in seedless oil palms, which could inhibit oleic acid accumulation. In contrast, the elevated expression of *FabB* and *LACS6* genes in the thin-shelled oil palms appeared to promote oleic acid accumulation in thin-shelled oil palms. The mid-developmental stage emerged as crucial for oleic acid synthesis, with genes such as *FabD*, *LACS6*, *BC*, *SAD*, *FabB*, and *FabI* exhibiting high expression, levels, lead to a significant increase in oleic acid content during this period. By the late developmental stage, oleic acid levels stabilized, with comparable levels observed in both seedless and thin-shelled varieties, This stabilization likely resulted from distinct regulatory mechanisms involving the expression of *LACS9* and *LACS6*. These findings provide a theoretical foundation for selecting germplasm with high oleic acid content and breeding oil palm varieties enriched in unsaturated fatty acids. Future research incorporating proteomic analyses will be essential to identify differentially accumulated enzymes and establish stronger connections between transcriptomic changes and metabolite profiles. Such approaches will offer deeper insights into the regulatory mechanisms governing oleic acid biosynthesis, enabling more precise strategies for metabolic engineering and crop improvement.

## Data Availability

The datasets presented in this study can be found in online repositories. The names of the repository/repositories and accession number(s) can be found in the article/**Supplementary Material**.
