# Supplementary material for: Transcriptional and metabolic analysis of oleic acid synthesis in seedless and tenera oil palm species
Source: Front Plant Sci. 2025 Feb 25;16:1557544. doi: 10.3389/fpls.2025.1557544 (PMC11893603; doi:10.3389/fpls.2025.1557544)
Supplement: Supplementary file 1 [file DataSheet1.docx]

Supplementary Material.

##
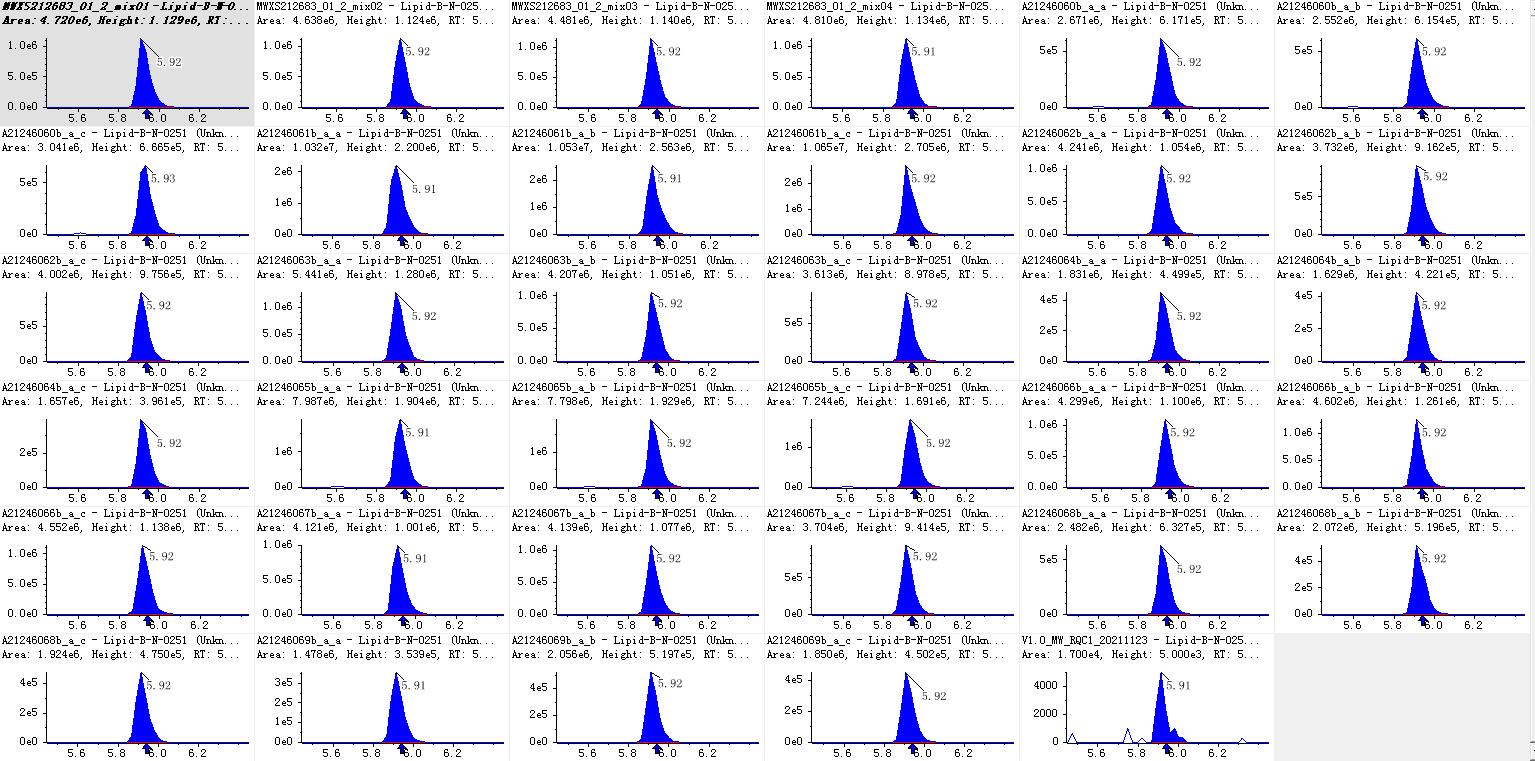
Supplementary Figure


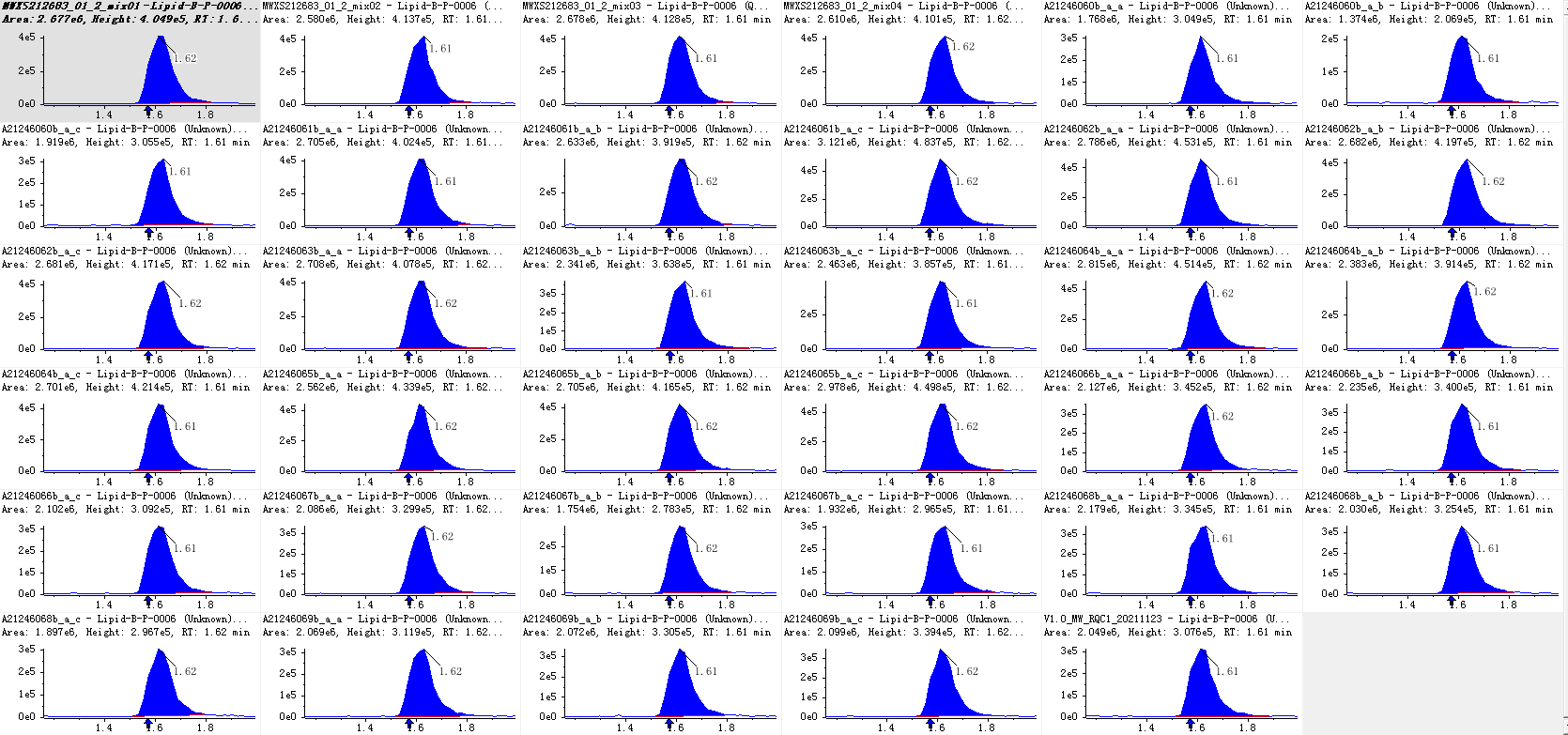


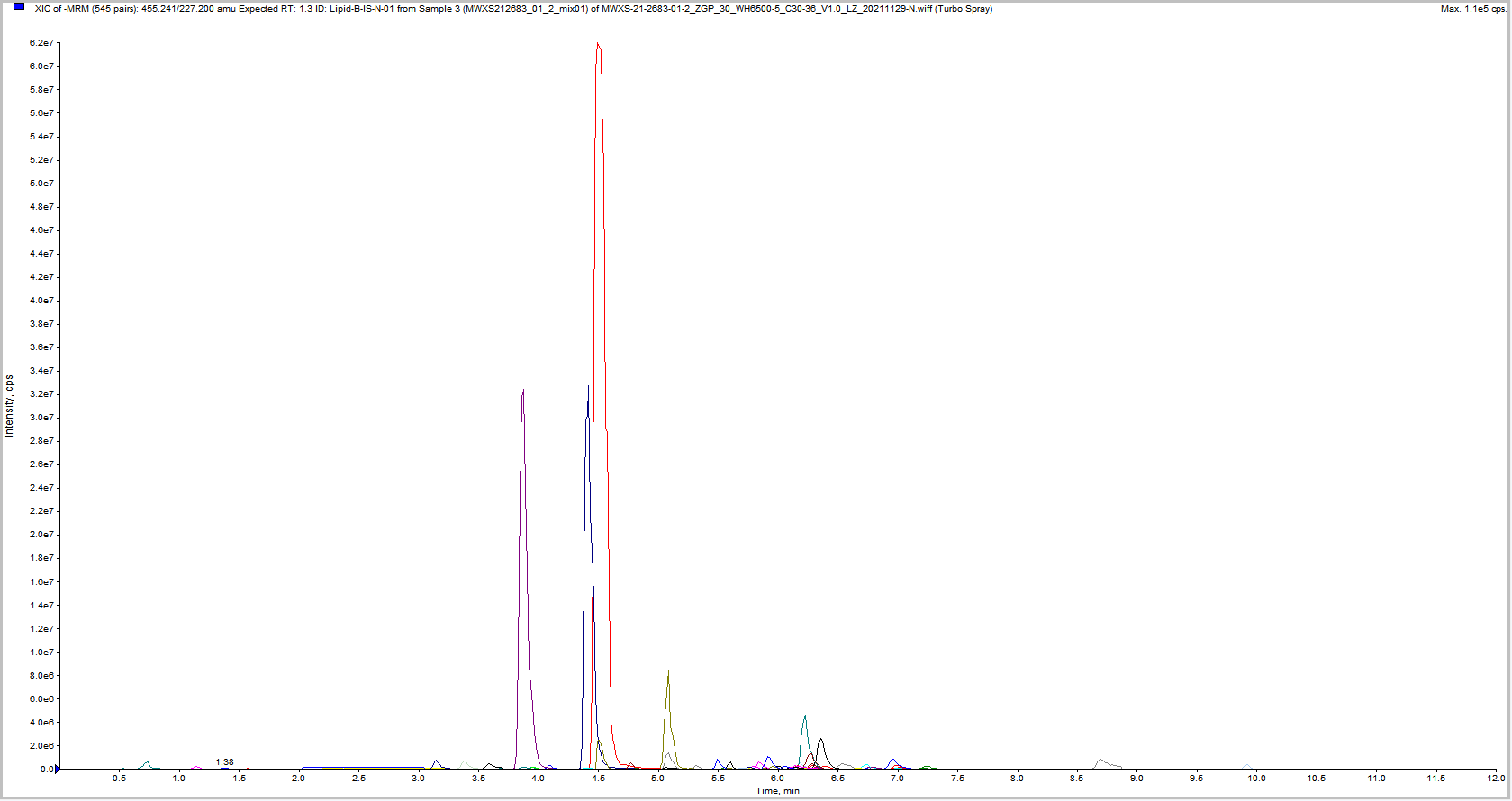
**Supplementary Figure S1.** :Correction chart for mass spectrometry integration of fatty acid metabolites


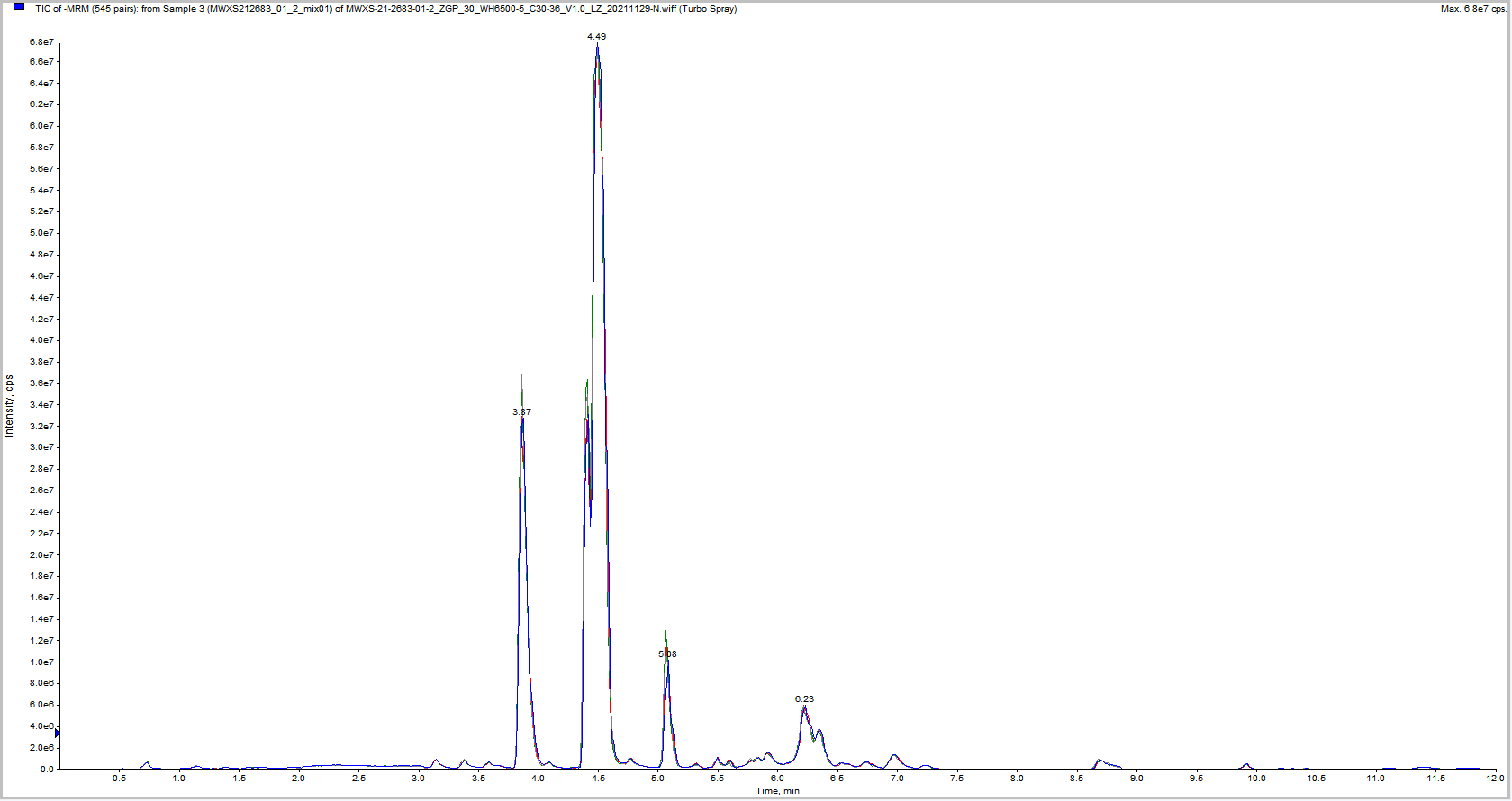

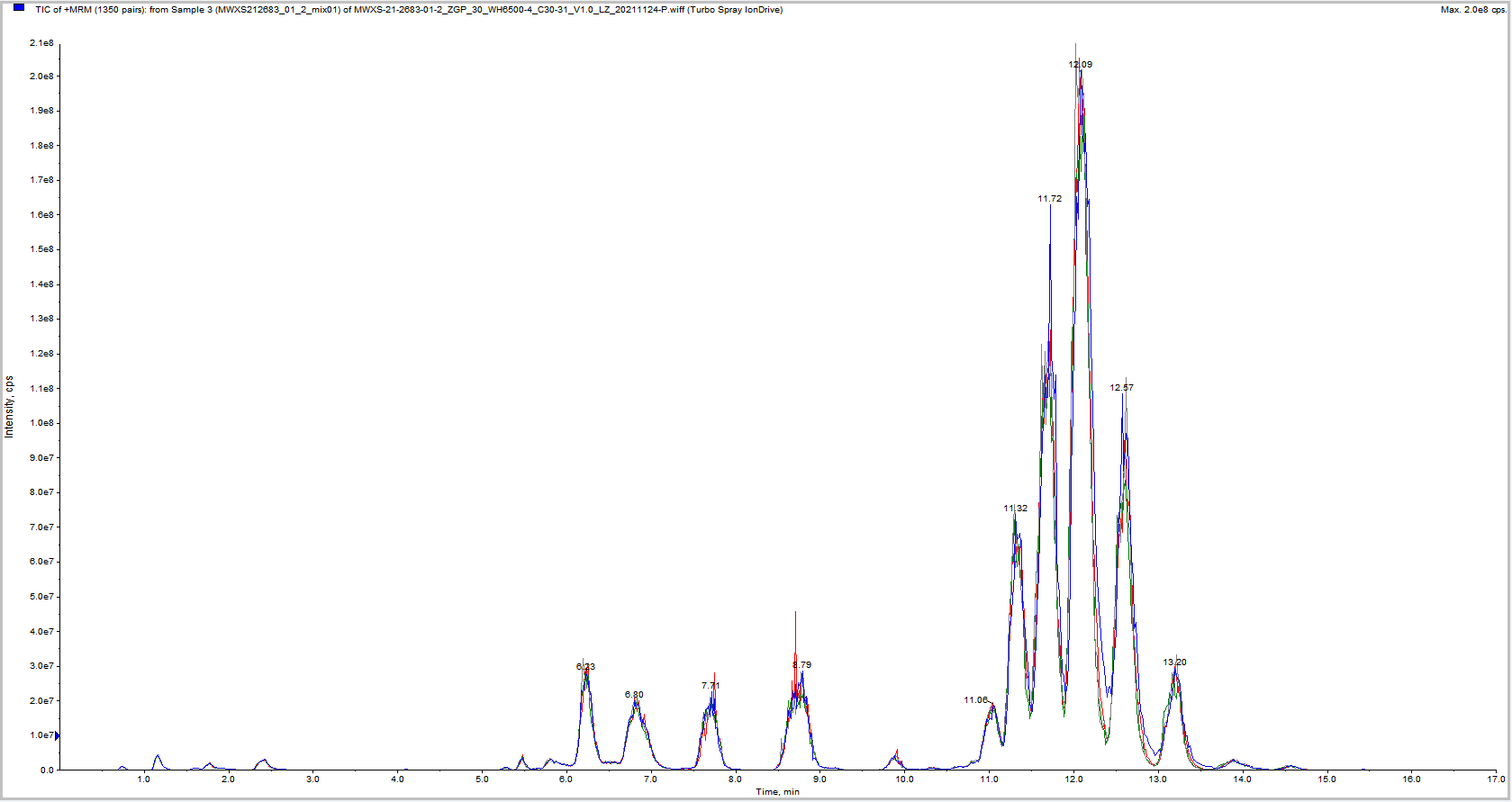

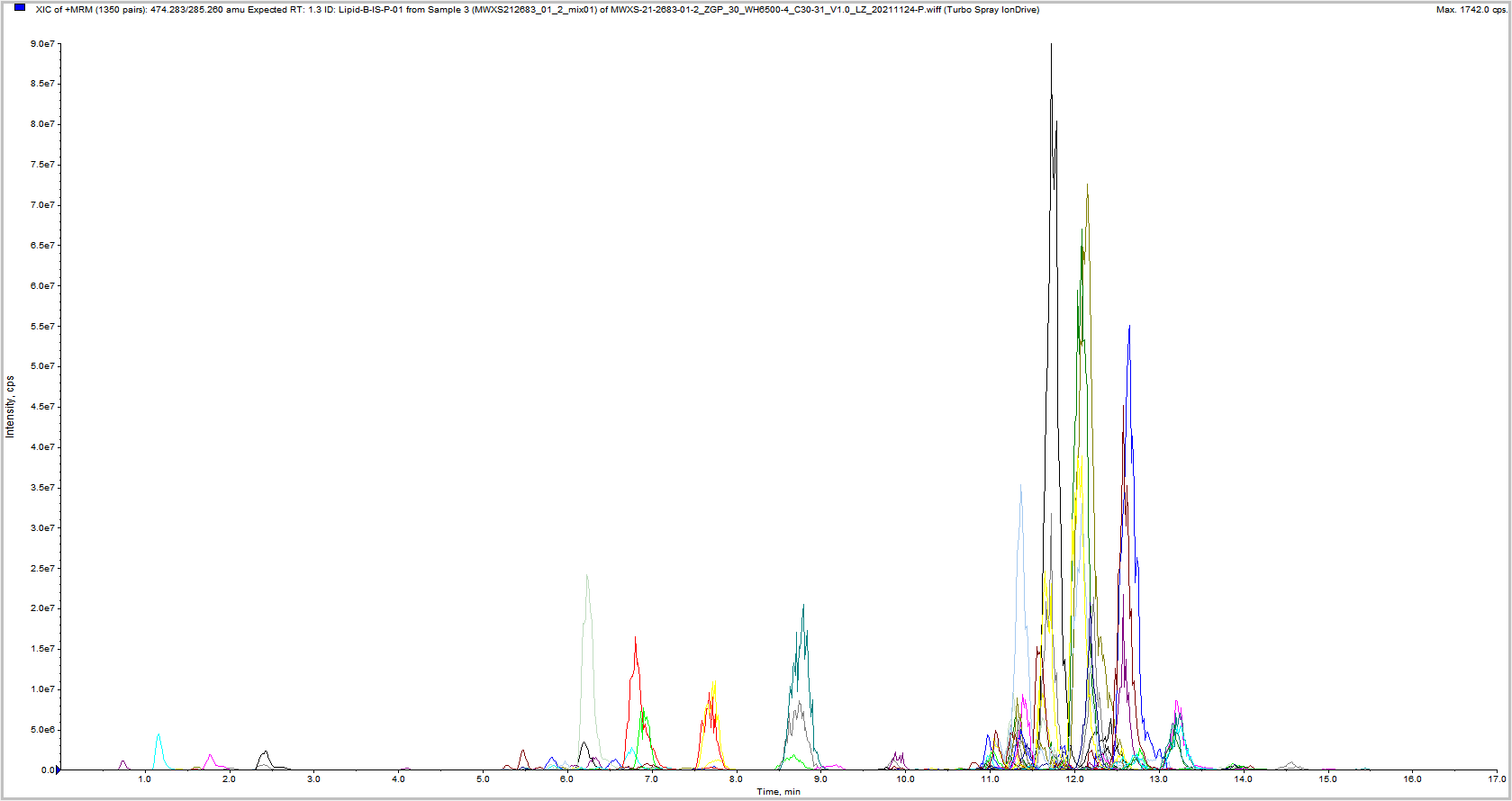


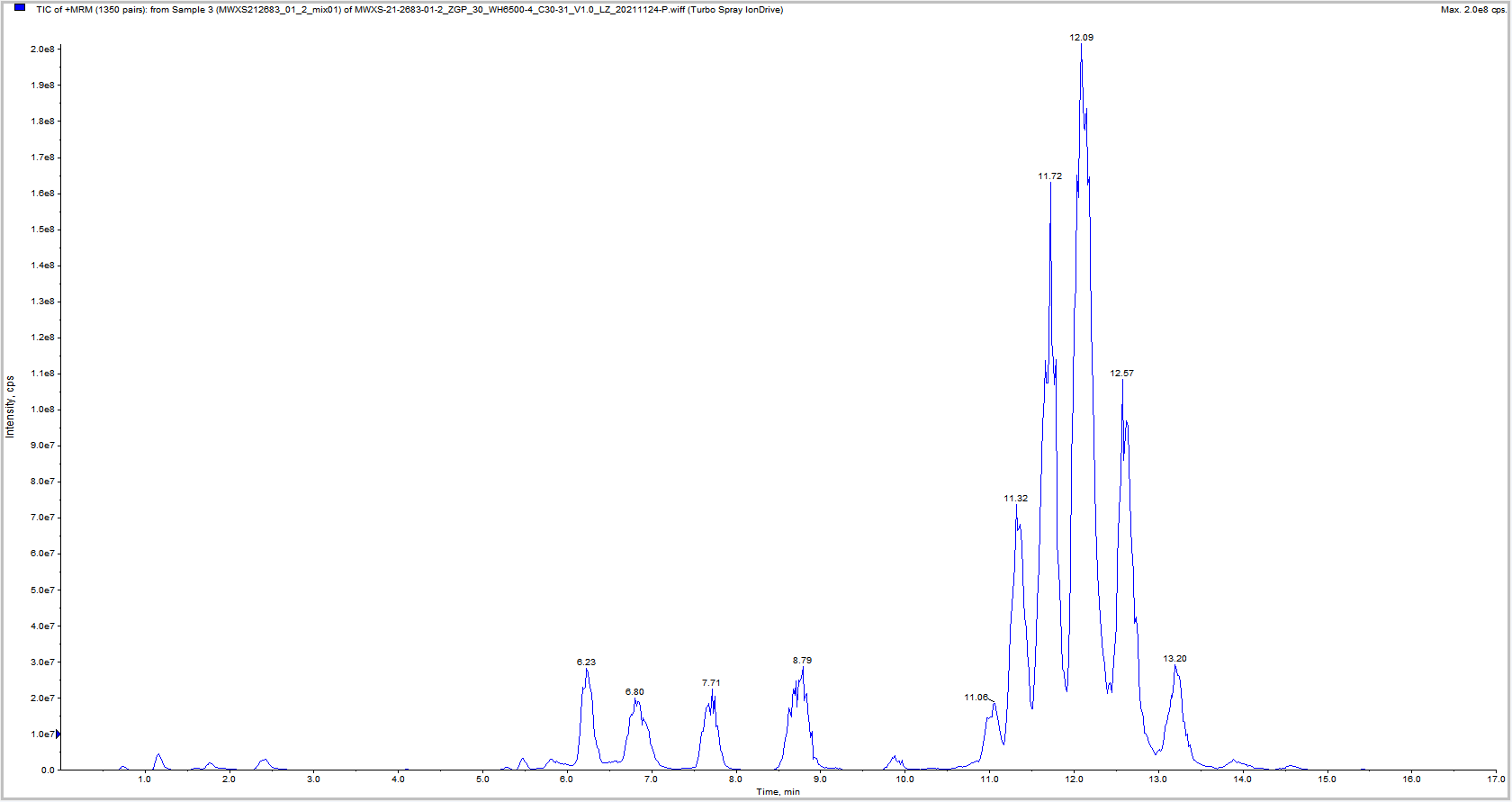

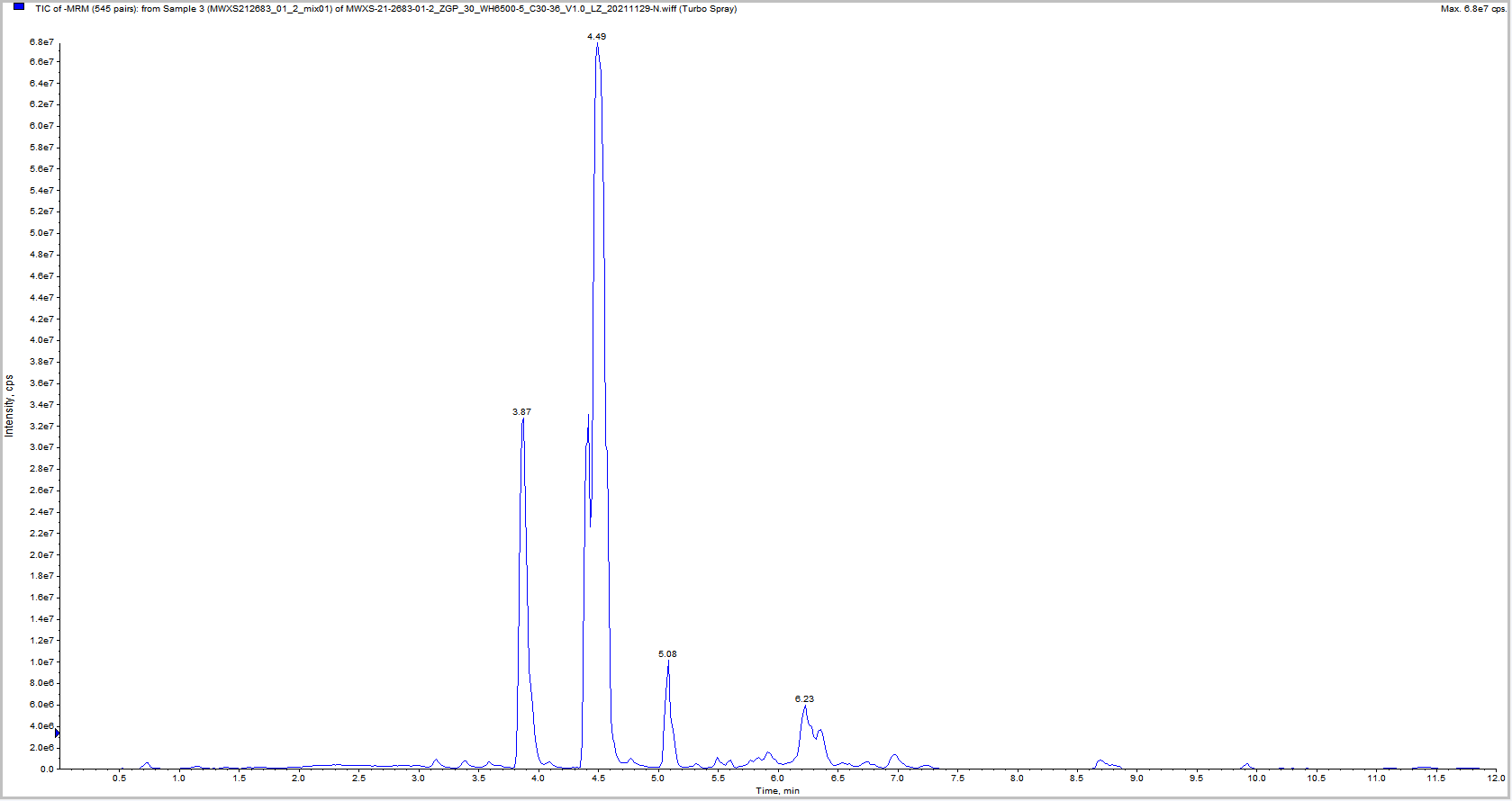


**Supplementary Figure S2.** The MRM model


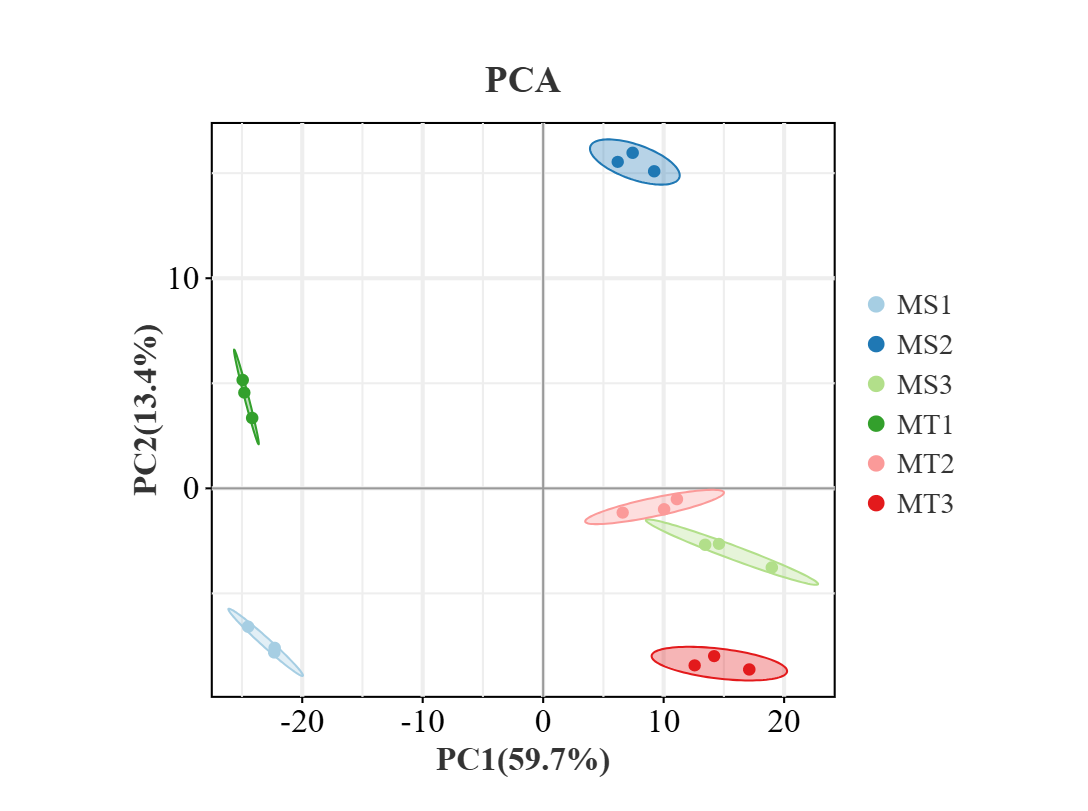


**Supplementary Figure S3**: Principal Component Analysis (PCA) score plot illustrating the distribution of metabolites based on their quantitative and qualitative profiles.
